# Supplementary material for: Causal interplay between lactose intolerance and gut microbiota: a combined bidirectional Mendelian randomization and in vivo validation study
Source: Front Nutr. 2026 Jun 1;13:1803337. doi: 10.3389/fnut.2026.1803337 (PMC13265576; doi:10.3389/fnut.2026.1803337)
Supplement: Supplementary file 1 [file Data_Sheet_1.zip › supplementary materials/Forward/leave one out cross validation/ebi-a-GCST90027757.finngen_R12_E4_LACTONAS.pdf]

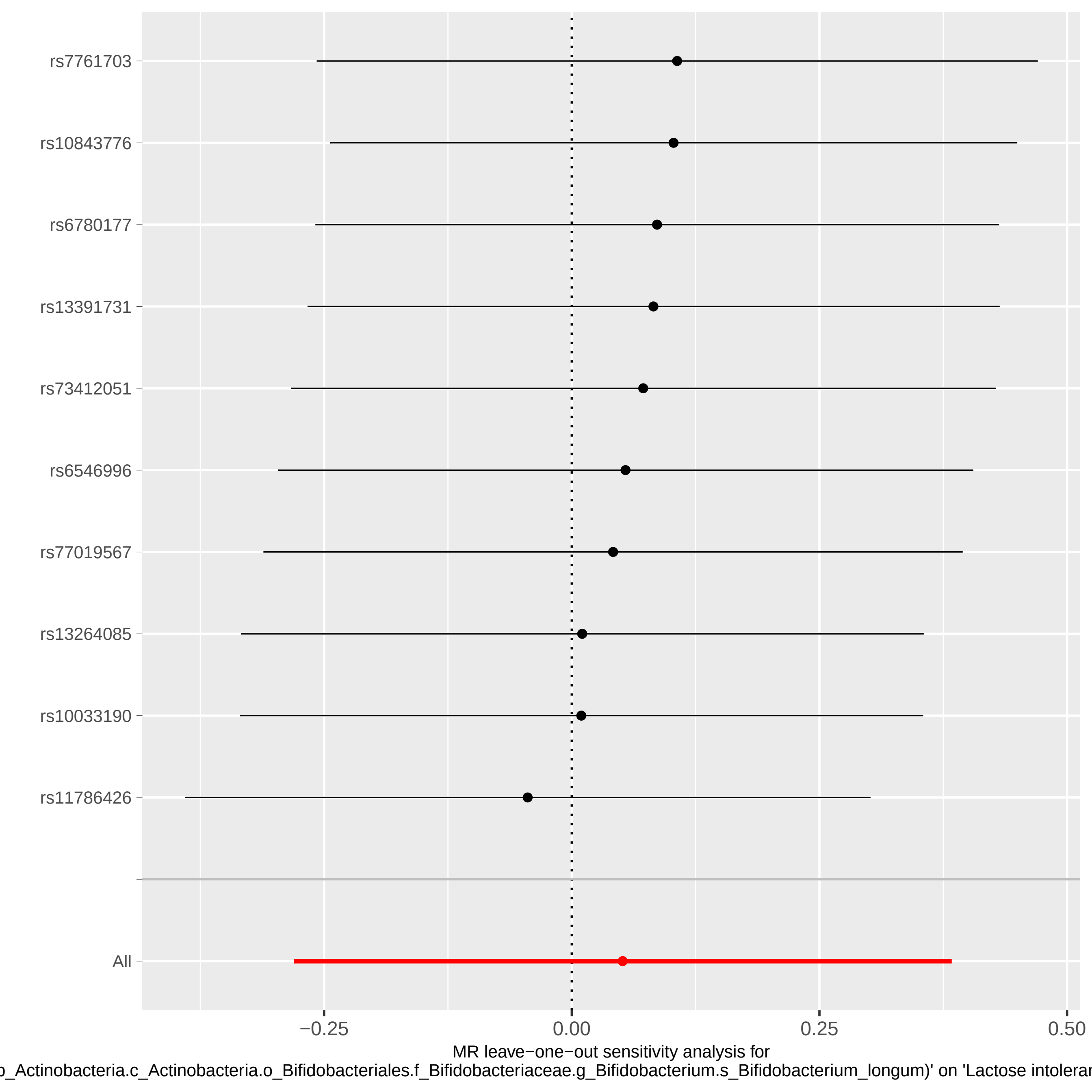

rs7761703
rs10843776
rs6780177
rs13391731
rs73412051
rs6546996
rs77019567
rs13264085
rs10033190
rs11786426
All
−0.25
0.00
0.25
0.50
MR leave−one−out sensitivity analysis for
p_Actinobacteria.c_Actinobacteria.o_Bifidobacteriales.f_Bifidobacteriaceae.g_Bifidobacterium.s_Bifidobacterium_longum)' on 'Lactose intoleran
